# Germinal center kinase-like kinase (GLK/MAP4K3) expression is increased in adult-onset Still's disease and may act as an activity marker

**DOI:** 10.1186/1741-7015-10-84

**Published:** 2012-08-06

**Authors:** Der-Yuan Chen, Huai-Chia Chuang, Joung-Liang Lan, Yi-Ming Chen, Wei-Ting Hung, Kuo-Lung Lai, Tse-Hua Tan

**Affiliations:** 1 Division of Allergy, Immunology and Rheumatology, Taichung Veterans General Hospital and Faculty of Medicine, National Yang Ming University, Taiwan; 2 School of Medicine, Chung Shan Medical University, Taichung, Taiwan; 3 Institute of Biomedical Science, National Chung Hsing University, Taichung, Taiwan; 4 Immunology Research Center, National Health Research Institutes, Zhunan, Taiwan; 5 Department of Pathology & Immunology, Baylor College of Medicine, Houston, Texas, USA

**Keywords:** Adult-onset Still's disease, GCK-like kinase (GLK, MAP4K3), mitogen-activated protein kinases (MAPKs), pathogenesis, Th17-related cytokines

## Abstract

**Background:**

Germinal center kinase-like kinase (GLK, also termed MAP4K3), a member of the MAP4K family, may regulate gene transcription, apoptosis and immune inflammation in response to extracellular signals. The enhanced expression of GLK has been shown to correspond with disease severity in patients with systemic lupus erythematosus. We investigated the role of GLK in the pathogenesis of adult-onset Still's disease, which shares some similar clinical characteristics with systemic lupus erythematosus.

**Methods:**

The frequencies of circulating GLK-expressing T-cells in 24 patients with active adult-onset Still's disease and 12 healthy controls were determined by flow cytometry analysis. The expression levels of GLK proteins and transcripts were evaluated in peripheral blood mononuclear cells by immunoblotting and quantitative PCR. Serum levels of T helper (Th)17-related cytokines, including IL-1β, IL-6, IL-17 and TNF-α, were measured by ELISA.

**Results:**

Significantly higher median frequencies of circulating GLK-expressing T-cells were observed in patients with adult-onset Still's disease (31.85%) than in healthy volunteers (8.93%, *P *<0.001). The relative expression levels of GLK proteins and transcripts were also significantly higher in patients with adult-onset Still's disease (median, 1.74 and 2.35, respectively) compared with those in healthy controls (0.66 and 0.92, respectively, both *P *<0.001). The disease activity scores were positively correlated with the frequencies of circulating GLK-expressing T-cells (r = 0.599, *P *<0.005) and the levels of GLK proteins (r = 0.435, *P *<0.05) or GLK transcripts (r = 0.452, *P *<0.05) in patients with adult-onset Still's disease. Among the examined Th17-related cytokines, elevated levels of serum IL-6 and IL-17 were positively correlated with the frequencies of circulating GLK-expressing T-cells and the levels of GLK proteins as well as transcripts in patients with adult-onset Still's disease. GLK expression levels decreased significantly after effective therapy in these patients.

**Conclusions:**

Elevated expression levels of GLK and their positive correlation with disease activity in patients with adult-onset Still's disease indicate that GLK may be involved in the pathogenesis and act as a novel activity biomarker of this disease.

## Background

Mitogen-activated protein kinases (MAPKs) comprise a family of cytoplasmic serine/threonine protein kinases that are involved in the regulation of key cellular processes including gene induction, cell proliferation, and inflammatory responses [1,2]. There are three major families of MAPKs, namely p38 MAPK, extracellular-regulated protein kinase and c-Jun N-terminal kinase (JNK) [3]. Wong *et al*. showed that activation of NF-κB, JNK and p38 MAPK play crucial roles in cytokine-mediated signaling pathways in T helper (Th) cells [4]. Moreover, the abnormal activation of intracellular MAPK upon IL-18- stimulation may account for hyperactivity of peripheral lymphocytes in systemic lupus erythematosus (SLE) [5]. A recent study demonstrated that activation of p38 MAPK contributes to Th17-cell effector function as well as the pathogenesis of Th17-mediated disease [6].

MAPK activation is mediated by upstream MAPK kinases, termed MAP2Ks (MKKs) and MAP3Ks (MKKKs). MAP4Ks, upstream kinases of MAP3Ks, likely regulate gene transcription, cell growth, apoptosis, and immune inflammation in response to extracellular signals [7,8]. Germinal center kinase-like kinase (GLK; also termed MAP4K3) is a member of the MAP4K family, which is a subfamily of the sterile 20 protein-like serine/threonine kinases [7]. GLK contains a conserved N-terminal kinase domain, a conserved C-terminal citron homology domain and proline-rich motifs in the middle portion [7]. Lam *et al*. identified GLK as a novel inducer of apoptosis [9], and apoptosis plays an important role in the pathogenesis of autoimmune diseases [10,11]. Our recent study showed an increased GLK expression that positively correlated with disease severity in patients with SLE [12]. In addition, previous studies showed that GLK-deficient mice were resistant to experimental autoimmune encephalomyelitis (EAE) [12], mediated mainly by Th17 cells [13].

Adult-onset Still's disease (AOSD) is an inflammatory disorder characterized by fever, rash, arthritis, variable multisystemic involvement, and an increase of acute phase reactants [14,15]. Our previous studies and other reports have shown that the levels of proinflammatory cytokines including IL-1β, IL-6, IL-18 and TNF-α are increased in patients with AOSD [16-19]. Furthermore, Th17 cells play an important role in the pathogenesis of AOSD [20]. These observations and a significant association of GLK with lupus disease activity [12] lead us to hypothesize that GLK may play a role in the pathogenesis of AOSD, which shares partial clinical manifestations with SLE. However, there is no data concerning GLK expression in AOSD.

In this study, we investigated whether GLK and Th17-related cytokines were involved in the pathogenesis of AOSD. The associations of GLK expression with disease activity and clinical characteristics were examined in patients with AOSD. The changes in GLK expression during longitudinal follow-up of these patients were also studied.

## Methods

### Participants

Twenty-four consecutive patients visiting Taichung Veterans General Hospital, Taiwan, with active untreated AOSD (15 women and 9 men, mean age ±SD, 33.3 ±9.9 years) fulfilling the Yamaguchi criteria [21] were enrolled. Patients with infections, malignancies or other rheumatic diseases were excluded. The disease activity scores (range, 0 to 12) for each patient were assessed according to the criteria described by Pouchot *et al*. [22]. After an initial determination of the levels of circulating GLK-expressing T-cells and Th17-related cytokines, all patients with AOSD received corticosteroids and non-steroidal anti-inflammatory drugs. The disease-modifying anti-rheumatic drugs used were methotrexate (20 patients), hydroxychloroquine (18 patients), sulfasalazine (8 patients), and azathioprine (3 patients). Twelve age-matched healthy volunteers (eight women and four men, mean age 32.4 ±8.2 years) who had no rheumatic disease served as normal controls. Peripheral blood was collected using endotoxin-free heparinized vacuum tubes (KABI-ET; Chromogenix, Antwerp, Belgium) to avoid cytokine production during the interval between sampling and culture. The Ethics Committee of Clinical Research, Taichung Veterans General Hospital, approved this study (No. C10130) and written consent was obtained from all participants according to the Declaration of Helsinki.

### Quantitation of circulating GLK-expressing T-cells using flow cytometry analysis

Circulating GLK-expressing T-cells were quantified using flow cytometry analysis according to the technique described in a recent study [12]. Antibodies for GLK were generated by immunizing rabbits with individual peptides [12]. Briefly, peripheral blood mononuclear cells (PBMCs) were harvested, washed with cold PBS, and stained with the indicated antibodies for 30 min on ice. PBMCs were then treated with Golgi-stop (10 μg/mL of Brefeldin A, Sigma, Schnelldorf, Deisenhofen, Germany) without any other stimulation and then stained with anti-CD3-allophycocyanin -Cy7,, anti-CD4-pacidic blue and anti-CD8-peridinin chlorophyll protein cyanin 5.5 (all BD Pharmingen, San Diego, CA, USA ), at room temperature (RT). For intracellular staining, PBMCs were permeabilized in 200 μL Cytofix/Cytoperm buffer (BD Biosciences, San Diego, CA, USA) for 2 h and washed with Perm-Wash buffer. The pellet was incubated with 100 μL Reagent 2, saponin (Beckman Coulter, Brea, CA, USA) for 5 min at in the dark. Samples were washed twice with 0.1% BSA-PBS, and incubated with phycoerythrin (PE)-conjugated GLK-specific mAb (eBiosciences, San Diego, CA, USA) for 30 minutes at in the dark. An isotype control IgG1-PE (eBiosciences) was used for GLK staining at RT in the dark. After staining, the cells were washed and immediately analyzed using flow cytometry (Beckman Coulter). Lymphocytes were gated on the basis of forward and size scatter properties, and at least 10,000 CD3^+ ^cells were analyzed. Data were collected using FACSCanto II flowcytometer (BD Biosciences) and analyzed by FlowJo software.

### Western blotting for GLK expression

For immunoblotting analysis, samples of purified T-cell were performed as described in our recent study [12]. For GLK, an equal amount of cell extracts from each set of experiments were fractionated on 6% to 8% SDS-PAGE in running buffer (25 mM Tris, 192 mM glycine, 0.1% SDS). The gel was run at 90 V for 30 min then at 130 V until the blue dye front reached the bottom. The gel was transferred to polyvinylidene difluoride membrane in transfer buffer (50 mM Tris, 384 mM glycine, 20% methanol) at 21 V for 1 h with the Trans-Blot SD Semi-Dry Electrophoretic Transfer Cell (Bio-Rad, Hercules, CA, USA). The membranes were blocked with 5% BSA in Tris buffered saline with Tween (TBST) (150 mM NaCl, 20 mM Tris-HCl (pH 7.4), 0.1% Tween-20 ) at RT for 1 h then probed with Anti-GLK (1:1,000), which was generated by immunizing rabbits with the appropriate peptide and anti-β-tubulin (1:1,000 T4026, Sigma, St. Louis, Missouri, USA) at 4°C overnight. The membranes were washed about three times with TBST, followed by incubation with peroxidase-conjugated secondary antibody (1:6,000) at RT for 1 h. The membranes of antibody reaction were washed three times with TBST and performed using the enhanced Immobilon Western Chemiluminescent HRP Substrate (WBKLS0500, Millipore, Billerica, Massachusetts, USA), exposed with a MegaCam 810 scientific grade CCD camera (UVP, LLC, Upland, CA, USA). The relative expression level of GLK protein was normalized to β-tubulin, and values were expressed relative to the control.

### Quantitative PCR for GLK expression

PBMCs were immediately isolated from venous blood using Ficoll-Paque PLUS (GE Healthcare Biosciences, Uppsala, Sweden) density gradient centrifugation. Total cellular RNA was obtained from PBMCs by the guanidinium isothiocyanate method [23] and was quantified by spectrophotometry at 260 nm. A 2.5 μg RNA aliquot was reverse transcribed with 200 U of Moloney murine leukemia virus reverse transcriptase (Fermentas, Thermo Fisher Scientific Inc.,Glen Burnie Maryland,USA) according to standard procedures. GLK mRNA expression levels were determined by quantitative PCR (qPCR) assay supplied in a TaqMan PCR Core Reagent Kit (Applied Biosystems, Foster City, CA, USA). Primers specific for GLK and the internal control glyceraldehydes-3-phosphate dehydrogenase (GAPDH) were obtained from Applied Biosystems. The purity of PCR products was assessed by dissociation curve plots. To standardize mRNA levels of GLK, transcript levels of the housekeeping gene GAPDH were also determined in parallel for each sample. The relative expression level of GLK was calculated with comparative threshold cycle (Ct) method and evaluated by:

$$2^{-\Delta \Delta \text{Ct}},\Delta \Delta \text{Ct = Patient(C}{\text{t}}_{\text{GLK}\;\text{gene}}\text{ - C}{\text{t}}_{\text{GAPDH}}\text{) - Mean}\;\text{of}\;\text{controls(C}{\text{t}}_{\text{GLK}\;\text{gene}}\text{ - C}{\text{t}}_{\text{GAPDH}}\text{)}\text{.}$$

### Determination of serum levels of soluble IL-2 receptor and Th17-related cytokines

Serum soluble IL-2 receptor (sIL-2R) levels were determined using an ELISA kit (Cellfree; Endogen Inc., Woburn, MA, USA). Serum levels of IL-1β, IL-6, IL-17A and TNF-α were determined in patients with AOSD and in healthy controls (HCs) using ELISA according to the manufacturer's instructions (eBiosciences).

### Statistical analysis

Results are presented as the mean ±SD or median (interquartile range). The nonparametric Kruskal-Wallis test was used for between-group comparison of the frequencies of circulating GLK-expressing T-cells, the expression levels of GLK transcript and protein, and serum levels of Th17-related cytokines. When this test showed significant differences, then the exact *P-*values were determined using the Mann-Whitney U test. The correlation coefficient was calculated using the nonparametric Spearman's rank correlation test. Wilcoxon signed rank test was employed to compare the levels of circulating GLK-expressing T-cells and the expression levels of GLK during follow-up in patients with AOSD after effective therapy. A *P *<0.05 was considered significant.

## Results

### Clinical characteristics of patients with adult-onset Still's disease

As illustrated in Table 1, all 24 patients with active untreated AOSD had daily spiking fevers (≥39°C). Other common manifestations included evanescent rash (n = 20, 83.3%), sore throat (n = 17, 70.8%) and arthritis (n = 15, 62.5%). Lymphadenopathy and hepatosplenomegaly were noted in 10 (41.7%) and six (25.0%) patients, respectively. There were no significant differences in age on entry to this study or the proportion of women between the patients with AOSD and HCs.

**Table 1:** Demographic data and clinical characteristics of patients with adult-onset Still's disease and healthy controls

Characteristics	Adult-onset Still's disease (n = 24)	Healthy controls (n = 12)
Age at study entry (years)	33.3 ±9.9	32.4 ±8.2
Proportion of females	15 (62.5%)	8 (66.7%)
Fever (≥38°C)	24 (100%)	NA
Evanescent rash	20 (83.3%)	NA
Sore throat	17 (70.8%)	NA
Arthritis	15 (62.5%)	NA
Lymphadenopathy	10 (41.7%)	NA
Liver dysfunction^a^	10 (41.7%)	NA
Hepatosplenomegaly	6 (25.0%)	NA
Clinical activity score	5.83 ±1.34	NA
C-reactive protein (mg/dL)	3.3 ±0.4	NA
Ferritin levels (μg/L)	692.9 ±103.6	NA
Soluble IL-2R levels (pg/mL)	563.5 ±87.5	NA

Data are presented as mean ±SD or number (percentage). ^a^Liver dysfunction was defined as alanine aminotransferase level ≧40 IU/L. NA: not applicable; IL-2R: interleukin-2 receptor.

### Increased frequencies of circulating GLK-expressing T-cells in patients with adult-onset Still's disease

**Figure 1:**
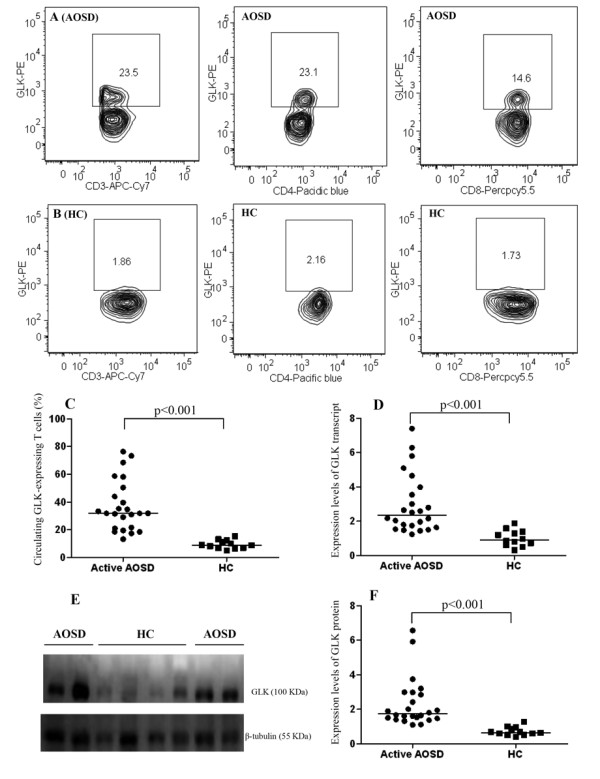
**The GLK expression levels in T cells from patients with adult-onset Still's diseases and healthy controls**. Representative examples of flow cytometry contour plots of intracellular GLK production in CD3^+ ^T-cells, CD4^+ ^T-cells, and CD8^+ ^T-cells were obtained from peripheral blood of **(A) **one patient with AOSD and **(B) **one healthy control. **(C) **The frequencies of circulating GLK-expressing CD3^+ ^T-cells were obtained from 24 patients with active AOSD and 12 HC. **(D) **The comparison in the relative expression levels of GLK transcript between patients with AOSD and HC. **(E) **Immunoblot analyses of GLK expression in the lysates of peripheral blood T cells from patients with AOSD and HC. **(F) **The comparison in the relative expression levels of GLK protein between patients with active AOSD and HC. Horizontal bar indicates median value. *P-value was determined by Mann-Whitney U test. AOSD: adult-onset Still's disease; GLK: germinal center kinase-like kinase; HC: healthy control.

Representative examples of flow cytometry contour plots of GLK expression in peripheral blood CD3^+ ^T-cells, CD4^+ ^T-cells and CD8^+ ^T-cells of one patient with active AOSD and one HC are shown in Figure 1A and 1B, respectively. Significantly higher median frequencies of circulating GLK-expressing CD3^+ ^T-cells were observed in patients with active AOSD (median = 31.85%, interquartile (IQ) range 21.21% to 48.84%) than in HCs (median = 8.93%, IQ range 6.81% to 12.08%; *P *<0.001, Figure 1C).

### Increased expression of GLK transcripts and proteins in patients with adult-onset Still's disease

As shown in Figure 1D, significantly greater fold increases in relative expression of GLK transcripts were observed in patients with active AOSD (median = 2.35, IQ range 1.66 to 3.88) than in HCs (median = 0.92, IQ range 0.63 to 1.37; *P *<0.001). Similarly, patients with active AOSD had increased expression of GLK in the lysates of purified T-cells determined by western blotting (Figure 1E). The relative expression levels of GLK proteins in patients with active AOSD (median = 1.74, IQ range 1.47 to 2.95) were significantly higher than those in controls (median = 0.66, IQ range 0.54 to 0.94; *P *<0.001, Figure 1F).

### Increased serum levels of Th17-related cytokines in patients with adult-onset Still's disease

**Figure 2:**
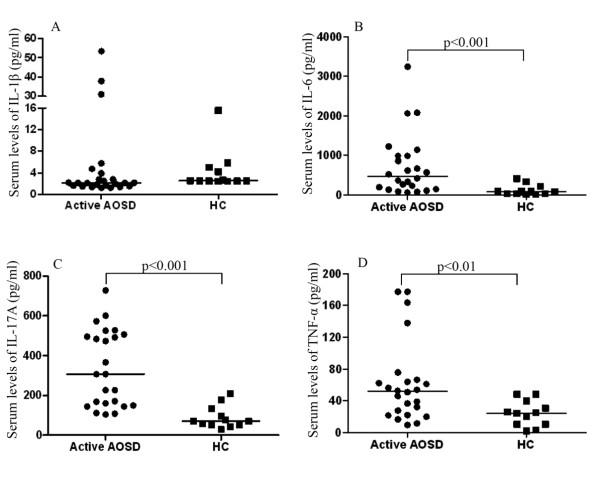
**The comparison in serum levels of Th17-related cytokines from patients with active adult-onset Still's disease and healthy controls**. **(A) **IL-1β, **(B) **IL-6, **(C) **IL-17A, and **(D) **TNF-α. Horizontal bar indicates median value. *P-value was determined by Mann-Whitney U test. AOSD: adult-onset Still's disease; HC: healthy control.

As shown in Figure 2, patients with active AOSD had significantly higher median levels of serum IL-6 (median = 474.81 pg/mL, IQ range 156.42 pg/mL to 987.55 pg/mL), IL-17A (median = 306.80 pg/mL, IQ range 152.17 pg/mL to 503.70 pg/mL) and TNF-α (median = 51.85 pg/mL, IQ range 23.63 pg/mL to 65.93 pg/mL) compared with those in HCs (median = 85.78 pg/mL, IQ range 31.13 pg/mL to 189.98 pg/mL, *P *<0.001 for IL-6; median = 70.90 pg/mL, IQ range 51.42 pg/mL to 124.53 pg/mL, *P *<0.001 for IL-17A; and median = 24.66 pg/mL, IQ range 10.50 pg/mL to 37.76 pg/mL, *P *<0.01 for TNF-α). However, there was no significant difference in serum IL-1β levels between patients with AOSD and HCs.

### Correlation between GLK expression and disease activity as well as cytokines in patients with adult-onset Still's disease

As illustrated in Table 2, the frequencies of circulating GLK-expressing CD3^+ ^T-cells were positively correlated with disease activity, including clinical activity scores, C-reactive protein levels, ferritin levels and serum levels of sIL-2R, which reflected T-cell activation in patients with AOSD. Similarly, the relative expression levels of GLK proteins and transcripts were positively correlated with clinical activity scores and sIL-2R levels in patients with AOSD. Among the Th17-related cytokines, GLK expression levels were positively correlated with serum levels of IL-6 and IL-17A. However, there was no significant correlation of GLK expression with clinical manifestations in our patients with AOSD (data not shown).

**Table 2:** The correlations between the frequencies of circulating GLK-expressing T-cells, the relative expression levels of GLK protein, GLK transcript and disease activity parameters as well as Th17-related cytokines in 24 patients with adult-onset Still's disease

	Circulating GLK-expressing T-cells (%)	Relative expression levels of GLK protein	Relative expression levels of GLK transcript
Clinical activity scores	0.599**	0.435*	0.452*
C-reactive protein (mg/dL)	0.455*	0.315	0.364
Ferritin (μg/L)	0.508*	0.296	0.318
Soluble IL-2R (pg/mL)	0.865***	0.569**	0.803***
IL-1β (pg/mL)	0.281	0.152	0.063
IL-6 (pg/mL)	0.822***	0.423*	0.547**
IL-17A (pg/mL)	0.787***	0.699***	0.740***
TNF-α (pg/mL)	0.295	0.177	0.310

GLK: germinal center kinase-like kinase; IL: interleukin; IL-2R: interleukin-2 receptor; TNF-α: tumor necrosis factor-alpha. *P <0.05, **P <0.005, ***P <0.001 obtained by the nonparametric Spearman's rank correlation test.

### Changes in the levels of GLK expression in patients with adult-onset Still's disease after effective therapy

**Figure 3:**
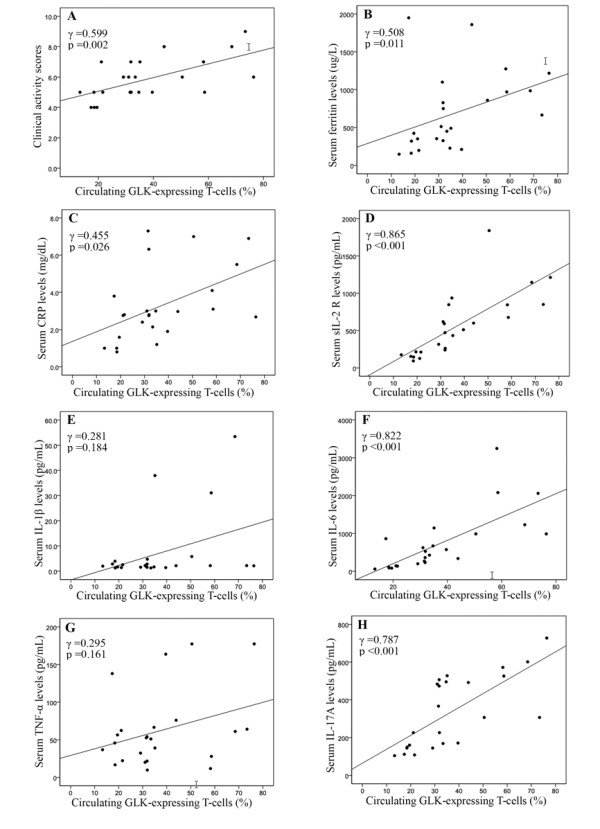
**Correlation between GLK expression and disease activity parameters as well as cytokines in patients with adult-onset Still's disease**. The correlation between the frequencies of circulating GLK-expressing T-cells and **(A) **disease activity score, activity parameters including **(B) **serum ferritin levels, **(C) **C-reactive protein levels and **(D) **soluble interleukin-2 receptor levels, and serum levels of cytokines including **(E) **IL-1β, **(F) **IL-6, **(G)**TNF-α and **(H) **IL-17A from 24 patients with adult-onset Still's disease. Correlation coefficients (γ) and *P*-value were obtained by the nonparametric Spearman's rank correlation test. AOSD: adult-onset Still's disease; CRP: C-reactive protein; GLK: germinal center kinase-like kinase; IL: interleukin; sIL-2R: soluble interleukin-2 receptor levels; TNF-α: tumor necrosis factor-alpha.

Twelve patients with AOSD were available for examination both at the active phase and at the remission phase. As shown in Figure 3, the percentages of circulating GLK-expressing T-cells and the relative expression levels (fold) of GLK proteins as well as transcripts were significantly decreased (mean ± standard error of the mean, 45.77 ±5.58% versus 20.11 ±2.53%; 3.01 ±0.49 versus 0.93 ±0.17; and 3.45 ±0.56 versus 1.21 ±0.38, respectively, all *P *<0.005), paralleling clinical remission and the decrease in serum levels of sIL-2R (747.8 ±131.8 pg/mL versus 229.1 ± 38.5 pg/mL, *P *<0.005) in patients with AOSD after effective therapy.

## Discussion

This study is the first investigation to demonstrate GLK overexpression in patients with active AOSD relative to HCs. The advent of flow cytometry analysis of intracellular signaling molecules [24] has greatly expanded the opportunities to study a single cell in heterogeneous cell populations. In the present study, CD3^+ ^T-cells, including CD4 and CD8 subsets, demonstrated increased GLK expression in patients with active AOSD. Our results also showed significantly elevated frequencies of circulating GLK-expressing T-cells, which correlated with disease activity, including clinical activity scores and serum ferritin levels, in patients with AOSD. Moreover, a parallel decrease in GLK production with disease remission was found in these patients. These data concerning patients with AOSD were similar to the results of our recent study showing elevated levels of circulating GLK-expressing T-cells correlating with activity index in patients with SLE [12], suggesting that GLK overexpression plays an important role in AOSD pathogenesis, and is thus a potential activity marker of this disease. However, a large prospective study should be conducted to confirm the findings presented here.

To verify GLK expression at the protein and transcript levels in patients with AOSD, western blotting and qPCR for GLK expression were performed in peripheral blood lymphocytes from our patients with active untreated AOSD. We have demonstrated that the relative expression levels of GLK proteins and transcripts were significantly higher in our patients than in HCs. Moreover, the positive correlations between the frequencies of circulating GLK-expressing T-cells and the expression levels of GLK proteins in our study are consistent with the findings of previous studies showing that intracellular flow cytometry and western blotting are equivalent assays for measuring MAPK signaling status [25,26]. In addition, the expression levels of GLK proteins as well as transcripts were significantly correlated with clinical activity scores in our patients with AOSD. These data provide the first direct and robust evidence of GLK overexpression in the T-cells of patients with AOSD.

Accumulating evidence indicates that Th17 cells play an important role in the pathogenesis of both AOSD and SLE [20,27,28]. IL-6 synergizes with IL-1β to enhance the differentiation and generation of Th17 cells [29]. Th17 cells can secrete IL-17, a pleiotropic cytokine which participates in tissue inflammation by inducing the expression of proinflammatory cytokines and chemokines [30-32]. Our recent study showed that GLK-deficient mice are resistant to the development of EAE and showed decreased Th17 responses [12]. The results from *in vitro *T-cell differentiation assays also indicate that GLK plays a positive role in Th17 cell differentiation [12]. In the present study, the results revealed elevated serum levels of Th17-related cytokines, IL-6 and IL-17A, which were correlated with the expression levels of GLK in T-cells from patients with AOSD. Our data also support previous findings showing that MAPK pathway plays an important role in the regulation of Th17 cell function [33], and that IL-17 production is mediated by a MAPK-dependent mechanism [34]. In addition, inhibition of MAPK could suppress IL-17 production in Vogt-Koyanagi-Harada syndrome [35], and attenuate the Th17-mediated autoimmune disease EAE [36]. These observations suggest either GLK overexpression or MAPK signaling can participate in the production of Th17-related cytokines. However, there still exists the possibility that GLK upregulation might represent an epiphenomenon of inflammation rather than a primary event in the pathogenesis of AOSD.

**Figure 4:**
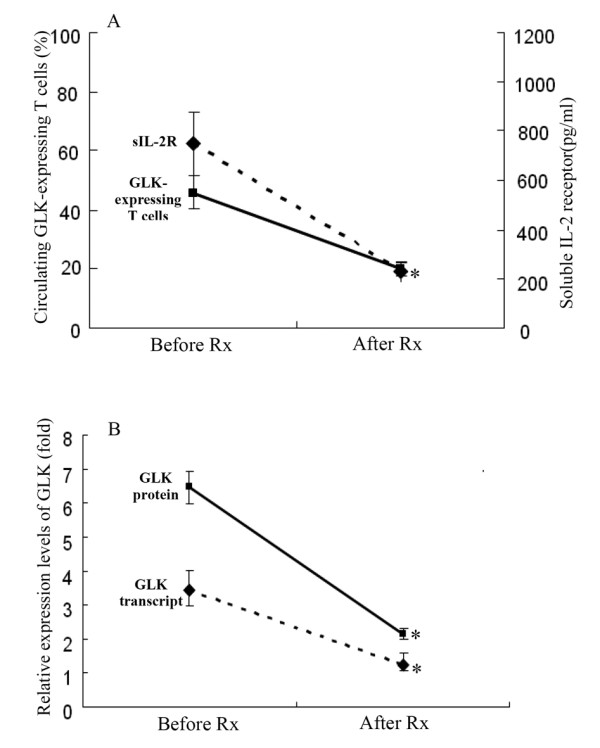
**Changes in the levels of circulating GLK-expressing T-cells, the expression levels of GLK proteins as well as transcripts, and serum levels of soluble interleukin-2 receptor in 12 patients with adult-onset Still's disease after effective therapy**. Data are presented as mean ± standard error of the mean. *P <0.005 versus before treatment, determined by the Wilcoxon signed rank test. AOSD: adult-onset Still's disease; GLK: germinal center kinase-like kinase; sIL-2R: soluble interleukin-2 receptor.

Our longitudinal follow-up of patients with AOSD showed a significant decrease in the levels of circulating GLK-expressing T-cells as well as the expression levels of GLK protein and transcript, paralleling the clinical remission and the decrease in inflammatory parameters after effective therapy (Figure 4). Our results support the hypothesis that inhibitors of more upstream MAPK signaling pathways, such as MAP2K (MKK3 or MKK6) and MAP3K (transforming growth factor activated kinase 1), can be a promising therapeutic modality for rheumatic diseases [37,38]. As an upstream MAPK, GLK could also be targeted as a potential therapeutic strategy by broadly inhibiting downstream MAPKs or several p38 isoforms [39,40]. Furthermore, upstream signaling molecules might be better targets than downstream molecules such as p38MAPK, blockade of which could result in considerable toxic effects [37,41,42].

There were some limitations in this study. Because biopsy tissue is difficult to obtain, we did not investigate the GLK expression in lesion specimens from patients with AOSD. Although some studies reported elevated IL-1β levels in AOSD and a substantial benefit of IL-1β receptor antagonist (anakinra) for treatment of inflammatory diseases [43,44], our results showed no significant difference in IL-1β levels between patients with AOSD and healthy volunteers. This discrepancy may result from differences in the detection methods or other unknown confounding factors undetected in this study. The lack of any significant association of GLK expression with clinical features may be owing to the small sample size in this clinically heterogeneous and uncommon disease.

## Conclusions

Our results revealed that GLK overexpression with increasing levels of Th17-related cytokines may be involved in the pathogenic mechanisms of AOSD. Our data add to the evidence supporting the association between GLK overexpression and a list of inflammatory diseases. We also showed that GLK expression levels were positively correlated with disease activity of AOSD, indicating that GLK might be a novel activity biomarker and a potential therapeutic target. Further investigations are required to confirm and extend this current finding.

## Abbreviations

AOSD: adult-onset Still's disease; BSA: bovine serum albumin; EAE: experimental autoimmune encephalomyelitis; GAPDH: glyceraldehydes-3-phosphate dehydrogenase; GLK: germinal center kinase-like kinase; HC: healthy control; IL: interleukin; IQ: interquartile; JNK: c-Jun N-terminal kinase; MAPKs: mitogen-activated protein kinases; NF: nuclear factor; PBMCs: peripheral blood mononuclear cells; PBS: phosphate-buffered saline; qPCR: quantitative polymerase chain reaction; SD: standard deviation; sIL-2R: soluble interleukin-2 receptor; SLE: systemic lupus erythematosus; TBST: Tris buffered saline with Tween; Th: T helper; TNF-α: tumor necrosis factor-alpha.

## Competing interests

The authors declare that they have no competing interests.

## Authors' contributions

All authors made substantive intellectual contributions to the present study and approved the final manuscript. D-YC conceived of the study, generated the original hypothesis, designed the study, acquired clinical data, performed data analysis, and drafted and revised the manuscript. T-HT conceived of the study, generated the original hypothesis, designed the study, performed statistical analysis, and revised the manuscript. H-CC, J-LL and Y-MC contributed equally on this work, and performed data acquisition and statistical analysis. W-TH and K-LL carried out clinical assessments on study participants.

## Pre-publication history

The pre-publication history for this paper can be accessed here:

http://www.biomedcentral.com/1741-7015/10/84/prepub
